# Evidence That Personal Genome Testing Enhances Student Learning in a Course on Genomics and Personalized Medicine

**DOI:** 10.1371/journal.pone.0068853

**Published:** 2013-07-23

**Authors:** Keyan Salari, Konrad J. Karczewski, Louanne Hudgins, Kelly E. Ormond

**Affiliations:** 1 Department of Genetics, Stanford University, Stanford, California, United States of America; 2 Department of Pediatrics, Division of Medical Genetics, Stanford University, Stanford, California, United States of America; 3 Stanford Center for Biomedical Ethics, Stanford University, Stanford, California, United States of America; Gentofte University Hospital, Denmark

## Abstract

An emerging debate in academic medical centers is not about the need for providing trainees with fundamental education on genomics, but rather the most effective educational models that should be deployed. At Stanford School of Medicine, a novel hands-on genomics course was developed in 2010 that provided students the option to undergo personal genome testing as part of the course curriculum. We hypothesized that use of personal genome testing in the classroom would enhance the learning experience of students. No data currently exist on how such methods impact student learning; thus, we surveyed students before and after the course to determine its impact. We analyzed responses using paired statistics from the 31 medical and graduate students who completed both pre-course and post-course surveys. Participants were stratified by those who did (*N* = 23) or did not (*N* = 8) undergo personal genome testing. In reflecting on the experience, 83% of students who underwent testing stated that they were pleased with their decision compared to 12.5% of students who decided against testing (*P* = 0.00058). Seventy percent of those who underwent personal genome testing self-reported a better understanding of human genetics on the basis of having undergone testing. Further, students who underwent personal genome testing demonstrated an average 31% increase in pre- to post-course scores on knowledge questions (*P* = 3.5×10^−6^); this was significantly higher (*P* = 0.003) than students who did not undergo testing, who showed a non-significant improvement. Undergoing personal genome testing and using personal genotype data in the classroom enhanced students' self-reported and assessed knowledge of genomics, and did not appear to cause significant anxiety. At least for self-selected students, the incorporation of personal genome testing can be an effective educational tool to teach important concepts of clinical genomic testing.

## Introduction

With the increasing affordability of personal genome testing (PGT), the incorporation of patient genotype data into the practice of medicine is becoming more pervasive. Multiple medical centers across the country have begun introducing genetic and genome-wide analysis to make pharmacogenetic testing available to patients [1], [2]. PGT companies offering direct-to-consumer (DTC) tests have empowered individuals to independently obtain their personal genomic profiles, which provide them with a view of their genetic risks for hundreds of diseases and atypical drug responses. Further, applications of genetic testing are expanding to pre-conception genetic screening [3], selection of embryos for *in vitro* fertilization [4], non-invasive screening for fetal chromosomal abnormalities [5], and the diagnosis of complex medical conditions [6].

Despite this expansion, most medical schools have not kept pace in providing state-of-the-art education in genetics and genomics to medical trainees [7]. Healthcare authorities and medical educators now agree that there is a strong need to train medical students and physicians to understand basic principles of genomics and to be able to interpret PGT results [8], [9]; however, there has been significant debate over the best educational models to deploy [10], [11]. Several institutions, including ours, have considered offering students the opportunity to undergo PGT themselves as part of an updated medical school genetics curriculum, with some institutions ultimately deciding against it [11]. At Stanford School of Medicine, after a school-wide task force rigorously evaluated potential risks and benefits, PGT was offered to students as part of a first-of-its-kind medical school elective course on genomics and personalized medicine, where students learn principles of genetics and genomics through a combination of interactive lectures and hands-on analysis of genomic data, using either their personal genotype data or publicly available datasets [10].

Given the novelty of this educational initiative, there was no data on how PGT impacts student learning and whether its use in the classroom enhances education. Therefore, we used a survey instrument administered before and after the course to examine associations between the use of PGT and student knowledge and attitudes about genomics. Based on previous evidence of the benefit of participatory learning in medical education [12], [13], [14], we hypothesized that the use of personal genome data in the classroom would improve knowledge and the learning experience for students.

## Materials and Methods

### Subjects

Subjects were medical and graduate students enrolled in an elective 8-week course on genomics and personalized medicine (Genetics 210; http://gene210.stanford.edu/) offered in the Summer 2010 quarter at Stanford School of Medicine. Forty-six students were enrolled in the course, and participation in this study was voluntary and anonymous.

### Genotyping

The course started with two weeks of instruction and class discussion led by a clinical geneticist (L.H.), a genetic counselor (K.E.O.), and a bioethicist/lawyer about the risks, benefits, uses, and limitations of PGT; these sessions provided the students with necessary background to provide informed consent should they proceed with PGT. At the end of the second week of instruction, students decided whether to personally undergo genotyping using the PGT services of either one of two PGT companies (23andMe or Navigenics). Of note, at the time of the course offering, 23andMe provided customers with their genotypes for all ∼600 K SNPs on their microarray while Navigenics provided genotypes for only the ∼300 SNPs used in their clinical reports. The subsequent six weeks of instruction included lectures and hands-on data analysis exercises on various topics related to human genetics, genomics, and personalized medicine (see course website for more details of the curriculum; http://gene210.stanford.edu/).

Each week students were led through classroom exercises to analyze various aspects of whole-genome single nucleotide polymorphism (SNP) data. A dataset comprising 12 diverse individuals from the HapMap project genotyped on Illumina HumanHap 650 K SNP microarrays were provided to all students. Students who underwent PGT were able to complete data analysis exercises using their personal genotype data, and students who did not undergo testing used publicly available genotype data from the 12 HapMap patients. A number of safeguards were implemented to ensure student privacy, confidentiality, and safety, including the provisioning of free genetic and medical counseling and mechanisms that students using their own data could ask questions if they had difficulty in resolving the class exercises without disclosing their genotype results [10].

### Survey Instrument

At the start and conclusion of the course, we electronically administered a survey that assessed student attitudes and knowledge about genomics and personalized medicine. The survey (extending the questionnaire developed by Ormond *et al*. [15]) included basic demographic information; assessed attitudes and knowledge about PGT; and solicited students' feedback on the experience of undergoing testing as it related to the class and their learning experience (**Methods S1**). Student attitudes were assessed either via yes/no questions or by asking for extent of agreement with statements on a 5-point Likert scale. Knowledge was assessed by both subjective and objective questionnaire items. For objective knowledge assessment, 6 multiple-choice questions and one free response question were asked; responses were scored blinded to subjects' genotyping status. Separate from the surveys presented in this study, students were also invited to participate in individual interviews discussing their experience (presented separately [16]). The Stanford University Institutional Review Board approved all study methodology.

### Data Analysis

We analyzed responses from students who completed both pre- and post-course surveys and attended at least 50% of the eight class sessions. Student responses to the pre- and post-course surveys were linked using a randomly assigned numeric code to maintain anonymity. We considered separating the students who underwent PGT before the course from those who underwent it during the course, but since preliminary statistical comparisons were underpowered to show differences, we elected to combine these groups in our study analysis. Student attitudes assessed on a 5-point Likert scale were collapsed and reported as the percentage of students who agree or strongly agree with the stem statement.

Paired pre-course and post-course responses were tested for change using paired non-parametric statistics (McNemar's test for binary response questions and the Wilcoxon signed-rank test for Likert items). Comparisons between responses of genotyped and non-genotyped students were made using Fisher's exact test for binary response questions and the Mann-Whitney *U*-test for Likert items. The change in student knowledge assessed by pre-course and post-course knowledge scores was evaluated by paired *t*-test. The difference in knowledge improvement between genotyped and non-genotyped students was assessed by Student's *t*-test.

## Results

Forty-three class participants completed the pre-course survey (93% response rate) and 34 class participants completed the post-course survey (74% response rate). We present data from 31 students who completed both pre- and post-course surveys, and attended at least 50% of the class sessions (67% of the course enrollees). Demographics from this study population are presented in **Table 1**; subjects were evenly split between genders, with slightly fewer medical versus non-medical trainees, and most frequently in their first year of training. Thirteen of the 17 students (76%) who indicated on the pre-course survey that they planned to undergo PGT did ultimately undergo testing. Seven students were initially unsure, of which 3 proceeded with testing. Another seven students had already undergone PGT prior to the course (all by 23andMe); these students indicated that they did not plan to undergo testing again and used their previously obtained data in the course. Due in part to a more limited genotype dataset provided by Navigenics, all 16 students who underwent PGT in the course did so via 23andMe. Thus, 23 students formed the genotyped group, and 8 students formed the non-genotyped group. There were no significant differences in demographics between the genotyped and non-genotyped groups (**Table 1**) or between class participants who completed the study and those who were lost to follow-up (**Table S1**).

**Table 1:** Subject characteristics.

	Genotyped^a^	Non-genotyped^a^	
Characteristics	*N* = 23	*N* = 8	*P* value^b^
Gender (female)	13 (56.5)	3 (37.5)	0.43
Program			0.21
Medical (MD, Clinical Resident/Fellow)	7 (30.4)	5 (62.5)	
Biomedical (PhD, Post-doctoral Fellow)	16 (69.6)	3 (37.5)	
Year in Program			0.27
1	11 (47.8)	2 (25.0)	
2	1 (4.3)	1 (12.5)	
3	3 (13.0)	3 (37.5)	
4+	8 (34.8)	2 (25.0)	
Previous personal genome testing	7 (30.4)	N/A	

a The number (and percentage) of subjects is reported.

b Fisher's exact test comparing genotyped and non-genotyped subjects.

### Pre and post-course attitudes toward personal genome testing

We surveyed student attitudes towards personal genome testing on pre- and post-course surveys (**Table 2**). Among genotyped students, 35–39% indicated that they would recommend PGT for a patient, with no significant difference between pre- and post-course responses. In contrast, among students who elected to not undergo testing, 50% indicated they would recommend PGT for patients before the course, but only 12% maintained this position after the course. Among proponents of PGT for patients, the most common reasons for support were to satisfy general curiosity about their genetic make-up (67%) and to see if a specific disease runs in their family or their DNA (56%). Those opposed to PGT for patients felt that such testing has limited clinical utility (82%), limited clinical validity (77%), individuals have a limited ability to understand and interpret their test results (68%), and not enough trained health care providers are available to help them interpret results (55%).

**Table 2:** Student attitudes toward personal genome testing.

	Genotyped group *N* = 23			Non-genotyped group *N* = 8			Genotyped *vs*. Non-genotyped
Question^a^	Pre	Post	*P* value^b^	Pre	Post	*P* value^b^	*P* value^c^
If you were to undergo PGT, would you share your results with a physician?	23 (100.0)	–		6 (75.0)	8 (100.0)		
If you were to undergo PGT, would you ask a health care provider for help in interpreting the results?	12 (52.2)	–		4 (50.0)	4 (50.0)		
Would you at this time recommend PGT for a patient?	9 (39.1)	8 (34.8)	1	4 (50.0)	1 (12.5)	0.25	0.38
Most people can accurately interpret their PGT results	0 (0.0)	1 (4.3)	**0.025**	0 (0.0)	0 (0.0)	1	0.38
PGT companies provide an accurate analysis and interpretation of genotype data	2 (8.7)	10 (43.5)	**0.02**	0 (0.0)	0 (0.0)	1	0.14
PGT companies should be regulated by the federal government	15 (65.2)	18 (78.3)	0.36	4 (50.0)	7 (87.5)	**0.037**	0.47

a For yes/no questions, the number (and percentage) of subjects responding yes is reported. For Likert items, the number (and percentage) of subjects who agreed or strongly agreed with the statement is reported.

b McNemar's test for binary response questions and Wilcoxon signed-rank test for Likert-scale items comparing pre- to post-course responses.

c Fisher's exact test for binary response questions and Mann-Whitney *U*-test for Likert-scale items comparing post-course responses between genotyped and non-genotyped groups.

At the start of the course, 100% of students felt that most people cannot accurately interpret their PGT results. Also, very few students felt that PGT companies provide an accurate analysis and interpretation of genotype data. However, after the course, significantly more students who underwent genotyping themselves believed that people could accurately interpret their results (*P* = 0.025, Wilcoxon signed-rank test) and that PGT companies provide an accurate analysis and interpretation (*P* = 0.02, Wilcoxon signed-rank test) (**Table 2**). In contrast, the non-genotyped group continued to feel that both patients and companies cannot accurately analyze or interpret PGT results. More than half of students felt that PGT companies should be regulated by the federal government, with significantly more non-genotyped students holding this opinion by the end of the course than at the beginning (*P* = 0.037, Wilcoxon signed-rank test; **Table 2**).

Notably, the majority of students (62%) indicated that they would undergo whole-genome sequencing in the future once it became affordable to them, including 50% of the students who chose not to undergo SNP-based genotyping at this time. Students overall felt PGT is an important educational topic, as 71% of students agreed or strongly agreed that it will likely play an important role in their future career.

### Knowledge of genetics and personal genome testing

We next examined students' reflections on their own knowledge of genetics and personal genome testing as well as that of practicing physicians (**Table 3**). Nearly all students felt that most physicians do not have enough knowledge to help individuals interpret PGT results, on both pre- and post-course surveys. Regarding their own knowledge, by the end of the course students in the genotyped group more strongly indicated that they understood the risks and benefits of using PGT services (*P* = 0.008, Mann-Whitney *U*-test) and that they knew enough about genetics to understand PGT results (*P* = 0.012, Mann-Whitney *U*-test) than students in the non-genotyped group (**Table 3**).

**Table 3:** Student perceptions of knowledge about genetics and personal genome testing.

	Genotyped group *N* = 23			Non-genotyped group *N* = 8			Genotyped *vs*. Non-genotyped
Question^a^	Pre	Post	*P* value^b^	Pre	Post	*P* value^b^	*P* value^c^
Most physicians have enough knowledge to help individuals interpret results of personal genome tests	0 (0.0)	1 (4.3)	0.41	0 (0.0)	0 (0.0)	0.77	0.96
I understand the risks and benefits of using PGT services	20 (87.0)	23 (100)	**0.003**	4 (50.0)	7 (87.5)	0.2	**0.008**
I know enough about genetics to understand PGT results	18 (78.3)	23 (100)	**0.005**	4 (50.0)	7 (87.5)	**0.02**	**0.012**
I have a better understanding of principles of human genetics on the basis of undergoing personal genotyping	–	16 (69.6)	–	–	–	–	–
Undergoing personal genotyping was an important part of my learning in GENE210	–	15 (65.2)	–	–	–	–	–
I would have learned just as much from GENE210 had I not undergone personal genotyping and only used publicly available genotype data	–	7 (30.4)	–	–	–	–	–
I would have learned more from GENE210 had I undergone personal genotyping instead of using publicly available geno type data	–	–	–	–	3 (37.5)	–	–

a The number (and percentage) of subjects who agreed or strongly agreed with each statement is reported.

b Wilcoxon signed-rank test comparing pre- to post-course responses.

c Mann-Whitney *U*-test comparing post-course responses between genotyped and non-genotyped groups.

Among genotyped subjects, 70% felt that they acquired a better understanding of principles of human genetics on the basis of undergoing PGT, and 65% felt that undergoing PGT was an important part of their learning in the course. Since all students were provided publicly available genotyping data from HapMap subjects to complete the in-class computer exercises, we specifically asked students to reflect on the use of personal versus publicly available genotype data. Only 30% of students who used personal genotype data felt that they would have learned just as much in the course had they not undergone testing and only used publicly available genotyping data. Conversely, a similar proportion of students in the non-genotyped group (37%) felt that they would have learned more in the course had they used personal genotype data instead of publicly available data.

**Figure 1:**
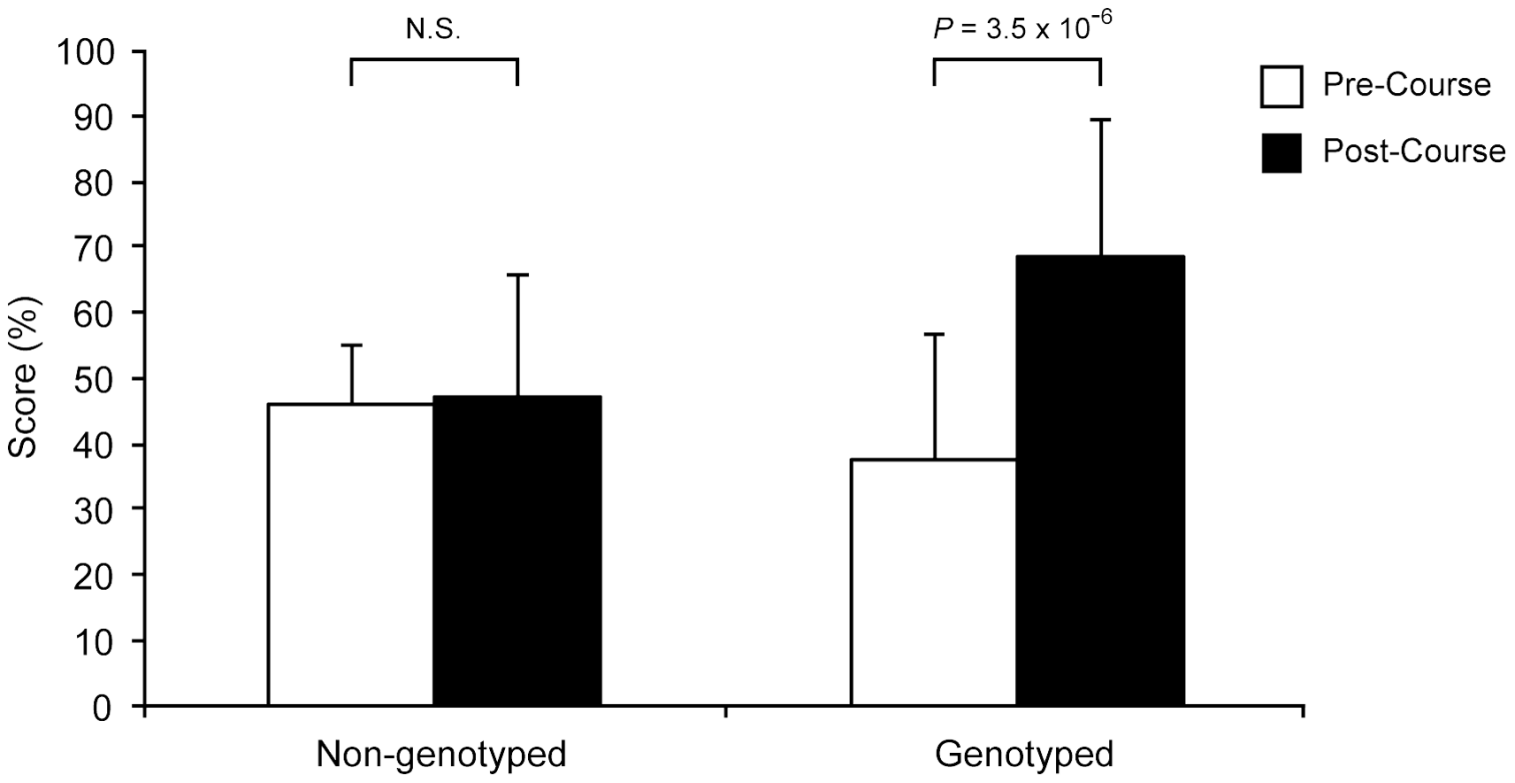
Student scores assessing knowledge of genomics. Knowledge scores of non-genotyped students on the post-course survey compared to the pre-course survey improved by an average of 1% (46% to 47%), while genotyped students demonstrated an average 31% improvement (38% to 69%). Bar graphs show mean (±S.D.) percentage score on knowledge questions.

To assess student knowledge of genetics and personal genome testing more objectively, we incorporated a short knowledge assessment in the pre- and post-course surveys, covering basic principles of genetics and clinical scenarios requiring the interpretation of PGT results (the same seven knowledge questions were asked on both surveys). At the start of the course, there was no significant difference between knowledge scores of students who did and students who did not undergo genotyping. However, by the end of the course we noted a significant improvement in knowledge scores only among students who underwent PGT (*P* = 3.5×10^−6^, paired *t*-test; **Figure 1**). Students in the non-genotyped group did not demonstrate significant improvement in their knowledge scores. The extent of improvement among genotyped students was significantly greater than that of non-genotyped students (31% *vs*. 1%, *P* = 0.002, Student's *t*-test).

### Genotyping process and experience

Students in the genotyped group most frequently reported having undergone testing due to general curiosity about their genetic make-up (100%), to help them understand principles of human genetics (57%), to help them understand what patients learn/experience (52%), and to see if a specific disease runs in their family or is in their DNA (52%). Non-genotyped students decided against testing due to concern that a for-profit company would have their DNA or genotype data (50%), concern that their data would not remain private (50%), and feeling that the information from SNP-based genotyping tests would not be useful (50%). Genotyped students were more likely than non-genotyped students to feel the course helped them understand a patient's experience in undergoing PGT (*P* = 0.00057, Mann-Whitney *U*-test; **Table 4** **)** and to be pleased with their decision to undergo genotyping (*P* = 0.00058, Mann-Whitney *U*-test; **Table 4**). Most of those who were not genotyped were neutral about their decision (75%).

**Table 4:** Student reflection on genotyping offer and experience.

Question^a^	Genotyped group*N* = 23	Non-genotyped group *N* = 8	
This course helped me understand what a patient's experience might be like if they chose to undergo personal genotyping	23 (100.0)	4 (50.0)	**0.00057**
Pleased with decision regarding personal genotyping	19 (82.6)	1 (12.5)	**0.00058**
Experienced anxiety when deciding whether to undergo personal genotyping	3 (13.0)	4 (50.0)	**0.0087**
Experienced anxiety when awaiting PGT results	3 (13.0)	–	
Experienced anxiety after receiving PGT results	2 (8.7)	–	
The opportunity to ask healthcare professional (e.g. genetic counselor, medical geneticist, or other physicians) for help in interpreting the results is an important component to a personal genotyping offer	21 (91.3)	7 (87.5)	0.23

a The number (and percentage) of subjects who agreed or strongly agreed with each statement is reported.

b Mann-Whitney *U*-test comparing post-course responses between genotyped and non-genotyped groups.

When deciding whether to undergo PGT, only 13% of students who ultimately underwent testing reported experiencing anxiety, compared to 50% of students who did not undergo testing (*P* = 0.0087, Mann-Whitney *U*-test; **Table 4**). Few students who elected to undergo testing reported anxiety while awaiting their test results (13%) or after receiving the results (8.7%). Nearly all students in both the genotyped and non-genotyped groups agreed that the opportunity to ask healthcare professionals for help in interpreting test results was an important component to the PGT offer (**Table 4**).

### Effect of genotyping on behavior

When asked prior to the course, 100% of genotyped students and 75% of non-genotyped students indicated a willingness to share their PGT results with their physician (**Table 2**); most indicated they would do so only if they discovered they were at an elevated risk for a condition. Although students indicated a strong willingness to share, significantly fewer (approximately half in each group) indicated they would ask a healthcare professional for help interpreting the results. After actually undergoing PGT, even fewer students reported that they had already asked (13%) or were planning to ask (13%) a healthcare professional for help interpreting their test results; the remainder indicated that they do not plan to ask a healthcare professional for help.

All 23 genotyped students reported taking at least one action specifically on the basis of their PGT results (**Table S2**). Most frequently, students held discussions with their family about test results (78%) and to learn more about their family history (52%), and 70% of students performed internet searches to educate themselves on conditions for which they were found to be at risk. Three students reported changing their diet in a positive manner and 4 students reported contemplating positive diet, exercise, or smoking habits, but had not yet made changes. The actions were most commonly things that subjects reported already planning to do or were actively doing (30%), but some actions had been previously attempted and students indicated that their PGT results moved them to try again (22%). In 30% of instances, they reported that PGT results moved them to contemplate and/or attempt various positive behavior changes.

### Course curriculum reflection

Among numerous safeguards built into the course, students who elected to undergo PGT did so privately with a PGT company, and course instructors never asked students whether they had undergone genotyping or for their raw genotype data. Course instructors also asked that students not disclose whether they had undergone genotyping. We asked students to reflect on the experience of using personal genotype data in the classroom and specifically, how well any concerns about privacy and confidentiality were addressed. Few students (2 genotyped, 1 non-genotyped) felt that the professors of the course knew whether they had undergone PGT; no student reported feeling at a disadvantage in the class as a result of this. Overall, most genotyped students (83%) felt they were easily able to go back and forth between their personal genotype data and the publicly available genotype data provided to them when working on the computer exercises, none felt required to divulge their genotype information in order to ask questions of the course professors, and 43% indicated they would have felt comfortable sharing their genotype data in order to ask questions of the course professors. Overall, all students felt that PGT should be made available to medical and graduate students as part of their genetics curriculum in some manner, but varied in their feelings towards whether it should be incorporated as an option in an elective course (61%) or core course (32%).

## Discussion

We report here the first study of educational outcomes in a course where students have the option to undergo personal genome testing. Overall, our results suggest that utilizing personal genotype data can augment the educational value of courses teaching concepts of genomics and personalized medicine. At the end of the course, genotyped and non-genotyped students alike viewed the option to undergo genotyping favorably, and PGT was incorporated into the curriculum in a manner that effectively maintained student safety, privacy, and confidentiality.

Most students who participated in this study took the course with the intention of undergoing PGT and adhered to their initial plan. However, the decision of a substantial fraction of students was influenced by the first two weeks of the course, which was spent discussing the risks, benefits, uses, and limitations of PGT services. We found that students were more likely to elect to undergo testing if they felt that they understood the risks and benefits of the test and enough about genetics to understand the results. Our data also suggest that students who experience anxiety during the decision-making process are more likely to decide against testing than students who do not. This is not surprising and reflects the self-selection process that is often seen in predictive genetic testing [17]. Together, these observations highlight the importance of a rigorous informed consent process prior to offering PGT, whether in a classroom setting or elsewhere.

Few genotyped students reported anxiety at any point in the process (deciding to undergo PGT, waiting for results, and after results were received), and none of the genotyped students reported regret with their decision on the post-course survey (4 weeks after receiving test results). These results are consistent with a recent report of subjects who underwent PGT with the Navigenics Health Compass [18], where such testing did not result in any measurable short-term changes in psychological health and over 90% of subjects experienced no test-related distress.

Students frequently cited gaining a better understanding of the patient experience as a reason that compelled them to undergo PGT. This parallels findings of a recent study of 137 Cleveland Clinic physicians who were offered PGT as a way to increase their familiarity with clinical genetics and PGT [19]. A majority of respondents in that study (77%) felt their personal experience pursuing PGT would benefit their patients directly by improving their ability to advise patients on the testing process and to relate to patients' experiences interpreting PGT results. Indeed, 100% of the genotyped students in our study reported that the course helped them understand the patient experience of undergoing PGT, compared to only half of non-genotyped students who indicated such an understanding. Despite this, most students still reported that, at this time, they would not recommend PGT for patients. However, students who underwent genotyping more often recommended it for patients than did students who did not undergo genotyping. These results are congruent with those of a recent study, in which primary care physicians currently offering PGT services as part of their practice were more likely to order the test for their patients if they felt well-informed about PGT and if they had undergone testing themselves [20].

The primary hypothesis of this study was that undergoing PGT would enhance the learning of students in the course. Since all students were provided publicly available genotype data from HapMap subjects to complete in-class computer exercises, we were able to specifically evaluate the educational utility of analyzing personal versus publicly available genotype data. Regardless of whether they used personal or public genotype data, by the end of the course nearly all students felt confident that they understood the risks and benefits of PGT and the underlying genetics required to understand PGT results. This stands in contrast to the previous study of students in our core medical school genetics course without PGT, in which only 20% of students felt they knew enough about genetics to understand PGT results by the end of the course [15]. While there are significant differences between the two courses (e.g., PGT is a smaller focus of the core course for medical students, and students in this study likely started with a greater level of understanding and interest based on prior coursework), our finding suggests that using genotype data of any sort (personal or public) to perform exercises on data analysis and interpretation enhances the learning experience of students.

We also found evidence specifically suggesting that PGT positively impacts learning for those students who self-select to undergo it. The majority of genotyped students felt they acquired a better understanding of the principles of human genetics on the basis of undergoing PGT and that the genotyping was an important part of their learning in the course. Substantiating these beliefs, genotyped students significantly improved their knowledge scores by an average of 31%, while non-genotyped students showed no significant difference in knowledge scores. The performance of non-genotyped students is similar to that described in the study of students in our core medical school genetics course without PGT, where only a modest improvement was noted between pre-course and post-course knowledge scores [15]. Together, these data suggest that some students derive greater educational benefit by undergoing PGT and using personal genotype data in the classroom than students who strictly use publicly available data or no data at all. As has been suggested in other educational contexts [12], [13], [14], analyzing and interpreting data with personal relevance may encourage students to be more engaged with the material, leading to greater understanding and retention of knowledge. For example, a recent report describes a genotyping exercise in a pharmacy class where 10 student volunteers provided DNA samples that were subjected to genotype analysis and presented to the class in the context of a genetic counseling session [13]. Students indicated in a survey that the exercise engaged them with the course content and would positively influence their ability to apply pharmacogenetic principles to patient care.

Undergoing PGT and interpreting test results also led some students to make or consider behavioral changes. Almost one-third of genotyped students indicated that due to elevated risks, they had already changed or were contemplating changes to their diet, exercise, or smoking habits. However, a longer-term qualitative study conducted on a small number of our students indicates that 6 months after receiving PGT results, none had taken significant behavioral actions [16], suggesting that early behavioral changes may not be sustained. These results mirror the recent study by Bloss *et al*.; while they found no significant change between baseline and follow-up in dietary fat intake or exercise behavior of subjects who underwent PGT with the Navigenics Health Compass, they also found that a substantial fraction of subjects contemplated behavioral changes or intended to undergo more medical tests [18]. The studies' differences may reflect differences in study population (mean age was 46.7 compared to our younger population of students in their mid-twenties), or more likely, length of follow-up (mean 5.6 months compared to 4 weeks in our study).

As an exploratory study of the first iteration of the course, this study has several limitations. The small sample size, self-selection of enrollees in an elective course, and single-institution setting of our study make broad generalizability of our results difficult. We also do not know the extent to which the educational benefits noted here extend to other types of learners, such as undergraduate students or practicing physicians, since our study was conducted primarily on medical and graduate students who expressed a specific interest in the topic of genotyping and personal genome testing. Finally, the survey instrument used in the study is not validated, and thus we cannot exclude the possibility that unclear wording in the questions may have affected some of our findings.

These limitations notwithstanding, our study represents the first line of evidence that the use of personal genome testing can enhance genetics education for at least a subset of learners. As personal genome testing becomes more widely-used in the classroom, future work should focus on conducting a randomized study where students who would like to undergo PGT are randomized to either undergo testing and work with their own genotype data or not undergo testing and work with publicly available genotype data. Such a study design would help control for any bias in educational outcomes resulting from self-selection and the results would be of great interest.

We believe it is imperative that medical school educators think creatively about how to incorporate education on this rapidly emerging area of medicine and science into their curricula. Our study finds that the interactive and participatory approach of using PGT in the classroom has the potential to increase students' knowledge and awareness of genetic testing. Although further study of its pedagogical utility is warranted, we believe when thoughtfully implemented, PGT can be used as a powerful and effective tool in genetics education.

## Supporting Information

Table S1 **Characteristics of subjects who completed study vs. lost to follow-up.**
(DOC)

Table S2 **Actions taken specifically as a result of receiving PGT results.**
(DOCX)

Methods S1 (PDF)
