# Supplementary material for: Evidence That Personal Genome Testing Enhances Student Learning in a Course on Genomics and Personalized Medicine
Source: PLoS One. 2013 Jul 23;8(7):e68853. doi: 10.1371/journal.pone.0068853 (PMC3720862; doi:10.1371/journal.pone.0068853)
Supplement: Table S1 — Characteristics of subjects who completed study vs. lost to follow-up. (DOC) [file pone.0068853.s001.doc]

**Table S1. Characteristics of subjects who completed study vs. lost to follow-up**

|  | **Completed Study** | **Lost to Follow-up** |  |
| --- | --- | --- | --- |
| **Characteristics** | **No. (%)** | **No. (%)** | ***P* value^a^** |
| Gender (female) | 16 (51.6) | 4 (25.0) | 0.12 |
| Program |  |  | 0.54 |
| Medical (MD, Clinical Resident/Fellow) | 12 (38.7) | 8 (50.0) |  |
| Biomedical (PhD, Post-doctoral Fellow) | 19 (61.3) | 8 (50.0) |  |
| Year in Program |  |  | 0.13 |
| 1 | 13 (41.9) | 11 (68.8) |  |
| 2 | 2 (6.5) | 2 (12.5) |  |
| 3 | 6 (19.4) | 2 (12.5) |  |
| 4+ | 10 (32.3) | 1 (6.3) |  |
| Previous personal genome testing | 7 (22.6) | 1 (6.3) | 0.23 |

^a^Fisher’s exact test comparing subjects who completed study and those lost to follow-up.
