# Supplementary material for: Evidence That Personal Genome Testing Enhances Student Learning in a Course on Genomics and Personalized Medicine
Source: PLoS One. 2013 Jul 23;8(7):e68853. doi: 10.1371/journal.pone.0068853 (PMC3720862; doi:10.1371/journal.pone.0068853)
Supplement: Table S2 — Actions taken specifically as a result of receiving PGT results. (DOCX) [file pone.0068853.s002.docx]

**Table S2. Actions taken specifically as a result of receiving PGT results**

| **Action** | **No. (%)** |
| --- | --- |
| Talked with family members about my genotyping results | 18 (78) |
| Performed internet research on the condition for which I am at risk | 16 (70) |
| Talked with family members to learn more about my family history | 12 (52) |
| Asked or planning to ask a healthcare professional for help interpreting results | 6 (26) |
| Contemplated diet, exercise, or smoking habits, but have not yet made changes | 4 (17) |
| Changed my diet in a positive manner due to elevated risks | 3 (13) |
| Increased my exercise habits | 2 (9) |
| Begun taking vitamins and/or homeopathic remedies | 1 (4) |
