# Supplementary material for: Evidence That Personal Genome Testing Enhances Student Learning in a Course on Genomics and Personalized Medicine
Source: PLoS One. 2013 Jul 23;8(7):e68853. doi: 10.1371/journal.pone.0068853 (PMC3720862; doi:10.1371/journal.pone.0068853)
Supplement: Methods S1 — (PDF) [file pone.0068853.s003.pdf]

Genetics 210 Pre-Course Survey

Randomized Code: ____________

Section I: Basic information

1. Please select your Stanford student affiliation

☐ MD student ☐ Graduate student ☐ MD/PhD ☐ Other ____________

2. Please enter the year you matriculated in the Stanford program indicated above:

____

3. Which of the following genetics courses have you taken in the past?

☐ Genetics 202
☐ Genetics 203
☐ Genetics 271
☐ None of the above

4. Have you undergone personal genotyping in the past?

☐ Yes ☐ No

If you answered **yes**, which company performed your personal genotyping?

☐ 23andMe
☐ Coriell Institute
☐ deCODEme
☐ DNA Direct
☐ Navigenics
☐ Pathway Genomics
☐ Other ________________________

5. Do you plan to undergo personal genotyping through the optional Stanford genotyping program in this course?

☐ Yes ☐ No ☐ Not sure

Genetics 210 Pre-Course Survey

Section II: Attitudes about personal genotyping

1. If you were to undergo personal genotyping, would you ask a health care provider for help in interpreting the results?

☐ Yes ☐ No

If yes, which type of healthcare provider (check all that apply):

☐ My primary physician (internist)
☐ A subspecialty physician (e.g. cardiologist, neurologist)
☐ A geneticist
☐ A genetic counselor
☐ It would depend on the results

2. If you were to undergo personal genotyping, would you share your results with your physician?

☐ Yes, regardless of my results
☐ Only if I were at high risk for something
☐ Only if I were not at high risk for something
☐ No

3. Would you, at this time, recommend a personal genotyping test for a patient?

☐ Yes ☐ No

If you answered **yes**, why (check all that apply)?

☐ To satisfy general curiosity about their genetic make-up
☐ To see if a specific disease runs in their family or is in their DNA
☐ To learn about their genetic make-up without going through a physician
☐ To inform family members of health risks
☐ Individuals have a right to know their genetic make-up if a service is available
☐ Other ________________________

If you answered **no**, why not (check all that apply)?

☐ Limited clinical validity
☐ Limited clinical utility
☐ Accuracy of genotype data
☐ Quality of data analysis / interpretation
☐ Array of SNPs / conditions tested
☐ Price
☐ Individuals have a limited ability to understand and interpret their test results

☐ Not enough trained health care providers to help them interpret results
☐ Other ________________________

4. I understand the risks and benefits of using personal genome testing services

☐ Strongly disagree ☐ Disagree ☐ Neither agree nor disagree ☐ Agree ☐ Strongly agree

5. I know enough about genetics to understand personal genome test results

☐ Strongly disagree ☐ Disagree ☐ Neither agree nor disagree ☐ Agree ☐ Strongly agree

6. Personal genomics will likely play an important role in my future career

☐ Strongly disagree ☐ Disagree ☐ Neither agree nor disagree ☐ Agree ☐ Strongly agree

7. Most physicians have enough knowledge to help individuals interpret results of personal genome tests

☐ Strongly disagree ☐ Disagree ☐ Neither agree nor disagree ☐ Agree ☐ Strongly agree

8. Most people can accurately interpret their personal genome test results

☐ Strongly disagree ☐ Disagree ☐ Neither agree nor disagree ☐ Agree ☐ Strongly agree

9. Personal genome testing companies provide an accurate analysis and interpretation of genotype data

☐ Strongly disagree ☐ Disagree ☐ Neither agree nor disagree ☐ Agree ☐ Strongly agree

10. Personal genome testing companies should be regulated by the federal government (i.e., by the Food and Drug Administration)

☐ Strongly disagree ☐ Disagree ☐ Neither agree nor disagree ☐ Agree ☐ Strongly agree

Genetics 210 Pre-Course Survey

Section III: Knowledge of personal genomic testing

Please select the most appropriate answer choice.

1. In an article published this year in the *New England Journal of Medicine*, whole-genome sequencing was performed on an individual with autosomal recessive Charcot-Marie-Tooth (CMT) disease. Mutations in the *SH3TC2* gene were identified as the causal mutations. The genotype of the proband was R954X/Y169H. The genetic basis of CMT discovered in this family is an example of:

(a) variable expressivity
(b) incomplete penetrance
(c) genetic mosaicism
(d) compound heterozygosity
(e) I do not know the answer to this question

2. A 34-year-old female of Northern European ancestry presents in your clinic with a strong family history of breast and ovarian cancer on both sides of the family. She brings along the results of her personal genomics testing by a direct-to-consumer (DTC) genetics company. Your patient's DTC genetics profile indicates she does *not* carry any of the 3 *BRCA1* or *BRCA2* mutations tested. Additionally, based on 3 other tested SNPs found to be associated with breast cancer in recent genome-wide association studies, her report reveals a risk of 9.1% compared to the average of 12.5%. What is the appropriate conclusion?

(a) The patient has below-average risk but should continue regular screening measures
(b) The patient has the same risk of breast cancer as the average woman of her ethnicity and should continue regular screening measures
(c) The patient has above-average risk and should consider getting her *BRCA1* gene sequenced
(d) The patient has above-average risk and should consider getting her *BRCA1* and *BRCA2* genes sequenced
(e) I do not know the answer to this question

3. The patient in question 3 is diagnosed with invasive breast cancer at age 38. Her cancer is most likely due to:

(a) an inherited founder mutation in *BRCA1*
(b) an inherited founder mutation in *BRCA2*
(c) an inherited non-founder mutation in *BRCA1*
(d) non-hereditary factors
(e) I do not know the answer to this question

Genetics 210 Pre-Course Survey

Questions 4-7 pertain to the following clinical scenario

Patient A is a 45-year-old white male who presents to your clinic with an unremarkable medical history. He has recently undergone personal genotyping by a DTC genetics company and comes to your office concerned about his risk of getting a heart attack.

According to the company, "numerous SNPs associated with one's chances of a heart attack have been found in the chromosomal region 9p21. [We] report your results for a SNP that is linked to the most strongly associated SNP in this region."

Your patient's genotype for this SNP (rs2383207) is G/G and the DTC genetics company calculates his risk of a heart attack to be elevated at 24.8%, compared to the average risk of 21.2% for men of European descent in the United States.

Based on individuals of European descent genotyped as part of the International HapMap project, the following table shows the genotype frequencies for rs2383207 in the population.

	**A/A**	**A/G**	**G/G**	**Total**
**Frequency**	0.183	0.583	0.233	1.00

4. Only taking into account his genotype at this one SNP, what is the **odds ratio** for Patient A getting a heart attack compared to the average man of European descent in the United States?

(a) 1.0
(b) 1.17
(c) 1.23
(d) 1.27
(e) I do not know how to calculate this

5. Patient A has two brothers under your medical care, B and C, both of whom also undergo personal genotyping. Patient B has genotype A/A at rs2383207 and his report indicates a risk of heart attack of 17.5% (i.e., P(D | "A/A") = 0.175). Patient C's genotype is A/G. Given your knowledge of the average risk for men of European descent, and of the genotype frequencies at rs2383207 in the European population, what would you expect patient C's risk to be? In other words, what is P(D | "A/G")?

(a) 17.9%
(b) 20.0%
(c) 21.0%
(d) 21.2%
(e) I do not know how to calculate this

Genetics 210 Pre-Course Survey

6. Based on their genetics reports, Patient B gloats that his risk for getting a heart attack is less than his brother, Patient A. You look at your medical charts and note that Patient B's total cholesterol level is 250 mg/dL, while Patient A's total cholesterol level is 180 mg/dL. Total cholesterol levels are classified as:

**Total cholesterol, mg/dL (mmol/L)**	
<200 (5.17)	Desirable
200 to 239 (5.17 to 6.18)	Borderline High
≥240 (6.20)	High

[Adult Treatment Panel III classification. *Circulation* 2002]

A prospective cohort study examining the relationship between baseline total cholesterol levels and myocardial infarctions among middle-aged men in the United States shows that the risk of MI in men with cholesterol levels ≥ 240 mg/dL is approximately 4 times that of men with cholesterol levels < 200 mg/dL [Stamler et al. *JAMA* 2000]. The mean cholesterol level of subjects in this study was 189.9 mg/dL. Thus, you can assume the odds ratio for an MI in men with cholesterol levels ≥ 240 mg/dL compared to the average man is 4.0.

Again, Patient B's genotype at rs2383207 is "A/A" and P(D | "A/A") = 0.175, and the average man has P(D) = 0.212. Integrate patient B's genetic risk with his environmental risk. Assuming each risk factor is statistically independent, what is patient B's risk of getting a heart attack, taking into account his genotype at rs2383207, and his cholesterol level of 250 mg/dL?

(a) 45.9%
(b) 52.6%
(c) 70.0%
(d) 84.8%
(e) I do not know how to calculate this

7. List at least 3 reasons why using one's genotype at rs2383207 to assess risk of a myocardial infarction can be problematic:

- rs238207 has modest odds ratio for MI
- Family history is stronger predictor than SNPs for genetic risk
- Only one SNP being used to assess risk, does not capture all of heritability
- rs2383207 is not the GWAS-identified SNP or disease-causing, and linkage disequilibrium blocks may differ from population to population
- Non-genetic risk factors play a large role in one's risk for MI

Genetics 210 Post-Course Survey (Not Genotyped)

Randomized Code: ____________

Section I: Basic information

1. How many class meetings of Genetics 210 did you attend?

☐ 0 ☐ 1 ☐ 2 ☐ 3 ☐ 4 ☐ 5 ☐ 6 ☐ 7 ☐ 8

Section II: Attitudes about personal genotyping

1. If you were to ever undergo personal genotyping in the future, would you ask a health care provider for help in interpreting the results?

☐ Yes ☐ No

If yes, which type of healthcare provider (check all that apply):

☐ My primary physician (internist)
☐ A subspecialty physician (e.g. cardiologist, neurologist)
☐ A geneticist
☐ A genetic counselor
☐ It would depend on the results

2. If you were to ever undergo personal genotyping in the future, would you share your results with your physician?

☐ Yes, regardless of my results
☐ Only if I were at high risk for something
☐ Only if I were not at high risk for something
☐ No

3. Would you, at this time, recommend a personal genotyping test for a patient?

☐ Yes ☐ No

If you answered **yes**, why (check all that apply)?

☐ To satisfy general curiosity about their genetic make-up
☐ To see if a specific disease runs in their family or is in their DNA
☐ To learn about their genetic make-up without going through a physician
☐ To inform family members of health risks
☐ Individuals have a right to know their genetic make-up if a service is available
☐ Other _________________________

If you answered **no**, why not (check all that apply)?

☐ Limited clinical validity
☐ Limited clinical utility
☐ Accuracy of genotype data
☐ Quality of data analysis / interpretation
☐ Array of SNPs / conditions tested
☐ Price
☐ Individuals have a limited ability to understand and interpret their test results
☐ Not enough trained health care providers to help them interpret results
☐ Other ________________________

4. How would you describe your understanding of preimplantation genetic diagnosis (PGD)?

☐ I have never heard of PGD
☐ I have heard of PGD, but I don't really understand what it is.
☐ I have heard of PGD, but I don't know enough about it to make a decision about whether or not I would consider it
☐ I have heard of PGD, and I know enough about it to make a decision about whether or not I would consider it

5. Under which of the following scenarios, if any, would you consider undergoing PGD? (check all that apply)

☐ You learned you had a highly penetrant cancer-associated mutation (e.g., BRCA mutation, MSH mutation)
☐ You learned you were a carrier for a Mendelian disorder (e.g., cystic fibrosis, Tay-Sach's disease)
☐ You learned you had a significantly increased risk for a complex disease (e.g. diabetes, heart disease)

6. For scenarios under which you *would not* consider undergoing PGD, which of the following most reflects your reason for not considering PGD?

☐ Low probability that your child would be affected by the disease
☐ Concern about danger to the embryo from biopsy required by PGD
☐ Ethical concerns about selecting embryos using genetics
☐ The high cost of PGD and *in vitro* fertilization
☐ Other ________________________

7. I understand the risks and benefits of using personal genome testing services

☐ Strongly disagree ☐ Disagree ☐ Neither agree nor disagree ☐ Agree ☐ Strongly agree

8. I know enough about genetics to understand personal genome test results

☐ Strongly disagree ☐ Disagree ☐ Neither agree nor disagree ☐ Agree ☐ Strongly agree

Genetics 210 Post-Course Survey (Not Genotyped)

9. Personal genomics will likely play an important role in my future career

☐ Strongly disagree ☐ Disagree ☐ Neither agree nor disagree ☐ Agree ☐ Strongly agree

10. Most physicians have enough knowledge to help individuals interpret results of personal genome tests

☐ Strongly disagree ☐ Disagree ☐ Neither agree nor disagree ☐ Agree ☐ Strongly agree

11. Most people can accurately interpret their personal genome test results

☐ Strongly disagree ☐ Disagree ☐ Neither agree nor disagree ☐ Agree ☐ Strongly agree

12. Personal genome testing companies provide an accurate analysis and interpretation of genotype data

☐ Strongly disagree ☐ Disagree ☐ Neither agree nor disagree ☐ Agree ☐ Strongly agree

13. Personal genome testing companies should be regulated by the federal government (i.e., by the Food and Drug Administration)

☐ Strongly disagree ☐ Disagree ☐ Neither agree nor disagree ☐ Agree ☐ Strongly agree

Genetics 210 Post-Course Survey (Not Genotyped)

Section III: Knowledge of personal genomic testing

Please select the most appropriate answer choice.

1. In an article published this year in the *New England Journal of Medicine*, whole-genome sequencing was performed on an individual with autosomal recessive Charcot-Marie-Tooth (CMT) disease. Mutations in the *SH3TC2* gene were identified as the causal mutations. The genotype of the proband was R954X/Y169H. The genetic basis of CMT discovered in this family is an example of:

(a) variable expressivity
(b) incomplete penetrance
(c) genetic mosaicism
(d) compound heterozygosity
(e) I do not know the answer to this question

2. A 34-year-old female of Northern European ancestry presents in your clinic with a strong family history of breast and ovarian cancer on both sides of the family. She brings along the results of her personal genomics testing by a direct-to-consumer (DTC) genetics company. Your patient's DTC genetics profile indicates she does *not* carry any of the 3 *BRCA1* or *BRCA2* mutations tested. Additionally, based on 3 other tested SNPs found to be associated with breast cancer in recent genome-wide association studies, her report reveals a risk of 9.1% compared to the average of 12.5%. What is the appropriate conclusion?

(a) The patient has below-average risk but should continue regular screening measures
(b) The patient has the same risk of breast cancer as the average woman of her ethnicity and should continue regular screening measures
(c) The patient has above-average risk and should consider getting her *BRCA1* gene sequenced
(d) The patient has above-average risk and should consider getting her *BRCA1* and *BRCA2* genes sequenced
(e) I do not know the answer to this question

3. The patient in question 3 is diagnosed with invasive breast cancer at age 38. Her cancer is most likely due to:

(a) an inherited founder mutation in *BRCA1*
(b) an inherited founder mutation in *BRCA2*
(c) an inherited non-founder mutation in *BRCA1*
(d) non-hereditary factors
(e) I do not know the answer to this question

Genetics 210 Post-Course Survey (Not Genotyped)

Questions 4-7 pertain to the following clinical scenario

Patient A is a 45-year-old white male who presents to your clinic with an unremarkable medical history. He has recently undergone personal genotyping by a DTC genetics company and comes to your office concerned about his risk of getting a heart attack.

According to the company, "numerous SNPs associated with one's chances of a heart attack have been found in the chromosomal region 9p21. [We] report your results for a SNP that is linked to the most strongly associated SNP in this region."

Your patient's genotype for this SNP (rs2383207) is G/G and the DTC genetics company calculates his risk of a heart attack to be elevated at 24.8%, compared to the average risk of 21.2% for men of European descent in the United States.

Based on individuals of European descent genotyped as part of the International HapMap project, the following table shows the genotype frequencies for rs2383207 in the population.

	**A/A**	**A/G**	**G/G**	**Total**
**Frequency**	0.183	0.583	0.233	1.00

4. Only taking into account his genotype at this one SNP, what is the **odds ratio** for Patient A getting a heart attack compared to the average man of European descent in the United States?

(a) 1.0
(b) 1.17
(c) 1.23
(d) 1.27
(e) I do not know how to calculate this

5. Patient A has two brothers under your medical care, B and C, both of whom also undergo personal genotyping. Patient B has genotype A/A at rs2383207 and his report indicates a risk of heart attack of 17.5% (i.e., P(D | "A/A") = 0.175). Patient C's genotype is A/G. Given your knowledge of the average risk for men of European descent, and of the genotype frequencies at rs2383207 in the European population, what would you expect patient C's risk to be? In other words, what is P(D | "A/G")?

(a) 17.9%
(b) 20.0%
(c) 21.0%
(d) 21.2%
(e) I do not know how to calculate this

Genetics 210 Post-Course Survey (Not Genotyped)

6. Based on their genetics reports, Patient B gloats that his risk for getting a heart attack is less than his brother, Patient A. You look at your medical charts and note that Patient B's total cholesterol level is 250 mg/dL, while Patient A's total cholesterol level is 180 mg/dL. Total cholesterol levels are classified as:

Total cholesterol, mg/dL (mmol/L)	
<200 (5.17)	Desirable
200 to 239 (5.17 to 6.18)	Borderline High
≥240 (6.20)	High

[Adult Treatment Panel III classification. *Circulation* 2002]

A prospective cohort study examining the relationship between baseline total cholesterol levels and myocardial infarctions among middle-aged men in the United States shows that the risk of MI in men with cholesterol levels ≥ 240 mg/dL is approximately 4 times that of men with cholesterol levels < 200 mg/dL [Stamler et al. *JAMA* 2000]. The mean cholesterol level of subjects in this study was 189.9 mg/dL. Thus, you can assume the odds ratio for an MI in men with cholesterol levels ≥ 240 mg/dL compared to the average man is 4.0.

Again, Patient B's genotype at rs2383207 is "A/A" and P(D | "A/A") = 0.175, and the average man has P(D) = 0.212. Integrate patient B's genetic risk with his environmental risk. Assuming each risk factor is statistically independent, what is patient B's risk of getting a heart attack, taking into account his genotype at rs2383207, and his cholesterol level of 250 mg/dL?

(a) 45.9%
(b) 52.6%
(c) 70.0%
(d) 84.8%
(e) I do not know how to calculate this

7. List at least 3 reasons why using one's genotype at rs2383207 to assess risk of a myocardial infarction can be problematic:

- rs238207 has modest odds ratio for MI
- Family history is stronger predictor than SNPs for genetic risk
- Only one SNP being used to assess risk, does not capture all of heritability
- rs2383207 is not the GWAS-identified SNP or disease-causing, and linkage disequilibrium blocks may differ from population to population
- Non-genetic risk factors play a large role in one's risk for MI

Genetics 210 Post-Course Survey (Not Genotyped)

Section IV: Attitudes and reflections on personal genotyping experience

1. For which of the following reasons did you decide to not undergo personal genotyping (check all that apply):

☐ Lack of curiosity about my genetic makeup
☐ Did not want to learn about specific diseases that run in my family or in my DNA
☐ Did not think the information from SNP-based genotyping tests would be useful
☐ Concerned that my data would not remain private
☐ Concerned that a for-profit company would have my DNA or genotype data
☐ Felt it could cause me additional or unnecessary worry or stress
☐ Other ________________________

Of these reasons, which was the *most* compelling (choose **one**):

☐ Lack of curiosity about my genetic makeup
☐ Did not want to learn about specific diseases that run in my family or in my DNA
☐ Did not think the information from SNP-based genotyping tests would be useful
☐ Concerned that my data would not remain private
☐ Concerned that a for-profit company would have my DNA or genotype data
☐ Felt it could cause me additional or unnecessary worry or stress
☐ Other ________________________

2. With respect to your feelings towards not undergoing personal genotyping, which of the following best reflects your beliefs:

☐ I have significant regret that I did not undergo it
☐ I have mild regret that I did not undergo it
☐ My feelings are neutral
☐ I am mildly pleased that I did not undergo it
☐ I am significantly pleased that I did not undergo it

3. What was the greatest benefit you experienced by not undergoing personal genotyping? (required comment box)

4. What was the greatest downside you experienced by not undergoing personal genotyping? (required comment box)

5. I experienced anxiety when deciding whether to undergo personal genotyping.

☐ Strongly disagree ☐ Disagree ☐ Neither agree nor disagree ☐ Agree ☐ Strongly agree

6. I believe the opportunity to seek counseling from a healthcare professional (e.g. genetic counselor, medical geneticist, or other physicians) is an important component to a personal genotyping offer.

Genetics 210 Post-Course Survey (Not Genotyped)

☐ Strongly disagree ☐ Disagree ☐ Neither agree nor disagree ☐ Agree ☐ Strongly agree

7. I would get my genome sequenced once it becomes affordable to me.

☐ Strongly disagree ☐ Disagree ☐ Neither agree nor disagree ☐ Agree ☐ Strongly agree

8. I would have learned more from Genetics 210 had I undergone personal genotyping instead of using publicly available genotype data.

☐ Strongly disagree ☐ Disagree ☐ Neither agree nor disagree ☐ Agree ☐ Strongly agree

9. This course helped me understand what a patient's experience might be like if they chose to undergo personal genotyping.

☐ Strongly disagree ☐ Disagree ☐ Neither agree nor disagree ☐ Agree ☐ Strongly agree

10. I felt that the professors knew whether I had undergone personal genotyping.

☐ Strongly disagree ☐ Disagree ☐ Neither agree nor disagree ☐ Agree ☐ Strongly agree

11. I felt that I was at a disadvantage in the class because the professors knew whether I had undergone personal genotyping.

☐ Strongly disagree ☐ Disagree ☐ Neither agree nor disagree ☐ Agree ☐ Strongly agree

12. With regards to the offer of personal genotyping as part of a curriculum, which of the following best reflects your beliefs:

☐ Personal genotyping should be required for all medical students and graduate students in the core curriculum (GENE 202/203)
☐ Personal genotyping should be made available on an optional basis for all medical students and graduate students in the core curriculum (GENE 202/203)
☐ Personal genotyping should be made available on an optional basis for medical students and graduate students in elective classes such as GENE 210
☐ Personal genotyping should not be made available as part of a course, but could be offered to students in an optional manner through other venues
☐ Personal genotyping should not be made available to students in any formal manner.

Genetics 210 Post-Course Survey (Genotype Data)

Randomized Code: ____________

Section I: Basic information

1. How many class meetings of Genetics 210 did you attend?

☐ 0 ☐ 1 ☐ 2 ☐ 3 ☐ 4 ☐ 5 ☐ 6 ☐ 7 ☐ 8

Section II: Attitudes about personal genotyping

1. Would you, at this time, recommend a personal genotyping test for a patient?

☐ Yes ☐ No

If you answered **yes**, why (check all that apply)?

☐ To satisfy general curiosity about their genetic make-up
☐ To see if a specific disease runs in their family or is in their DNA
☐ To learn about their genetic make-up without going through a physician
☐ To inform family members of health risks
☐ Individuals have a right to know their genetic make-up if a service is available
☐ Other ________________________

If you answered **no**, why not (check all that apply)?

☐ Limited clinical validity
☐ Limited clinical utility
☐ Accuracy of genotype data
☐ Quality of data analysis / interpretation
☐ Array of SNPs / conditions tested
☐ Price
☐ Individuals have a limited ability to understand and interpret their test results
☐ Not enough trained health care providers to help them interpret results
☐ Other ________________________

2. How would you describe your understanding of preimplantation genetic diagnosis (PGD)?

☐ I have never heard of PGD
☐ I have heard of PGD, but I don't really understand what it is.
☐ I have heard of PGD, but I don't know enough about it to make a decision about whether or not I would consider it
☐ I have heard of PGD, and I know enough about it to make a decision about whether or not I would consider it

3. Under which of the following scenarios, if any, would you consider undergoing PGD? (check all that apply)

Genetics 210 Post-Course Survey (Genotype Data)

☐ You learned you had a highly penetrant cancer-associated mutation (e.g., BRCA mutation, MSH mutation)
☐ You learned you were a carrier for a Mendelian disorder (e.g., cystic fibrosis, Tay-Sach's disease)
☐ You learned you had a significantly increased risk for a complex disease (e.g. diabetes, heart disease)

4. For scenarios under which you *would not* consider undergoing PGD, which of the following most reflects your reason for not considering PGD?

☐ Low probability that your child would be affected by the disease
☐ Concern about danger to the embryo from biopsy required by PGD
☐ Ethical concerns about selecting embryos using genetics
☐ The high cost of PGD and *in vitro* fertilization
☐ Other ________________________

5. I understand the risks and benefits of using personal genome testing services

☐ Strongly disagree ☐ Disagree ☐ Neither agree nor disagree ☐ Agree ☐ Strongly agree

6. I know enough about genetics to understand personal genome test results

☐ Strongly disagree ☐ Disagree ☐ Neither agree nor disagree ☐ Agree ☐ Strongly agree

7. Personal genomics will likely play an important role in my future career

☐ Strongly disagree ☐ Disagree ☐ Neither agree nor disagree ☐ Agree ☐ Strongly agree

8. Most physicians have enough knowledge to help individuals interpret results of personal genome tests

☐ Strongly disagree ☐ Disagree ☐ Neither agree nor disagree ☐ Agree ☐ Strongly agree

9. Most people can accurately interpret their personal genome test results

☐ Strongly disagree ☐ Disagree ☐ Neither agree nor disagree ☐ Agree ☐ Strongly agree

10. Personal genome testing companies provide an accurate analysis and interpretation of genotype data

☐ Strongly disagree ☐ Disagree ☐ Neither agree nor disagree ☐ Agree ☐ Strongly agree

11. Personal genome testing companies should be regulated by the federal government (i.e., by the Food and Drug Administration)

Genetics 210 Post-Course Survey (Genotype Data)

☐ Strongly disagree ☐ Disagree ☐ Neither agree nor disagree ☐ Agree ☐ Strongly agree

Genetics 210 Post-Course Survey (Genotype Data)

Section III: Knowledge of personal genomic testing

Please select the most appropriate answer choice.

1. In an article published this year in the *New England Journal of Medicine*, whole-genome sequencing was performed on an individual with autosomal recessive Charcot-Marie-Tooth (CMT) disease. Mutations in the *SH3TC2* gene were identified as the causal mutations. The genotype of the proband was R954X/Y169H. The genetic basis of CMT discovered in this family is an example of:

(a) variable expressivity
(b) incomplete penetrance
(c) genetic mosaicism
(d) compound heterozygosity
(e) I do not know the answer to this question

2. A 34-year-old female of Northern European ancestry presents in your clinic with a strong family history of breast and ovarian cancer on both sides of the family. She brings along the results of her personal genomics testing by a direct-to-consumer (DTC) genetics company. Your patient's DTC genetics profile indicates she does *not* carry any of the 3 *BRCA1* or *BRCA2* mutations tested. Additionally, based on 3 other tested SNPs found to be associated with breast cancer in recent genome-wide association studies, her report reveals a risk of 9.1% compared to the average of 12.5%. What is the appropriate conclusion?

(a) The patient has below-average risk but should continue regular screening measures
(b) The patient has the same risk of breast cancer as the average woman of her ethnicity and should continue regular screening measures
(c) The patient has above-average risk and should consider getting her *BRCA1* gene sequenced
(d) The patient has above-average risk and should consider getting her *BRCA1* and *BRCA2* genes sequenced
(e) I do not know the answer to this question

3. The patient in question 3 is diagnosed with invasive breast cancer at age 38. Her cancer is most likely due to:

(a) an inherited founder mutation in *BRCA1*
(b) an inherited founder mutation in *BRCA2*
(c) an inherited non-founder mutation in *BRCA1*
(d) non-hereditary factors
(e) I do not know the answer to this question

Genetics 210 Post-Course Survey (Genotype Data)

Questions 4-7 pertain to the following clinical scenario

Patient A is a 45-year-old white male who presents to your clinic with an unremarkable medical history. He has recently undergone personal genotyping by a DTC genetics company and comes to your office concerned about his risk of getting a heart attack.

According to the company, “numerous SNPs associated with one's chances of a heart attack have been found in the chromosomal region 9p21. [We] report your results for a SNP that is linked to the most strongly associated SNP in this region.”

Your patient’s genotype for this SNP (rs2383207) is G/G and the DTC genetics company calculates his risk of a heart attack to be elevated at 24.8%, compared to the average risk of 21.2% for men of European descent in the United States.

Based on individuals of European descent genotyped as part of the International HapMap project, the following table shows the genotype frequencies for rs2383207 in the population.

	A/A	A/G	G/G	Total
**Frequency**	0.183	0.583	0.233	1.00

4. Only taking into account his genotype at this one SNP, what is the **odds ratio** for Patient A getting a heart attack compared to the average man of European descent in the United States?

(a) 1.0
(b) 1.17
(c) 1.23
(d) 1.27
(e) I do not know how to calculate this

5. Patient A has two brothers under your medical care, B and C, both of whom also undergo personal genotyping. Patient B has genotype A/A at rs2383207 and his report indicates a risk of heart attack of 17.5% (i.e., P(D | “A/A”) = 0.175). Patient C’s genotype is A/G. Given your knowledge of the average risk for men of European descent, and of the genotype frequencies at rs2383207 in the European population, what would you expect patient C’s risk to be? In other words, what is P(D | “A/G”)?

(a) 17.9%
(b) 20.0%
(c) 21.0%
(d) 21.2%
(e) I do not know how to calculate this

Genetics 210 Post-Course Survey (Genotype Data)

6. Based on their genetics reports, Patient B gloats that his risk for getting a heart attack is less than his brother, Patient A. You look at your medical charts and note that Patient B's total cholesterol level is 250 mg/dL, while Patient A's total cholesterol level is 180 mg/dL. Total cholesterol levels are classified as:

Total cholesterol, mg/dL (mmol/L)	
<200 (5.17)	Desirable
200 to 239 (5.17 to 6.18)	Borderline High
≥240 (6.20)	High

[Adult Treatment Panel III classification. *Circulation* 2002]

A prospective cohort study examining the relationship between baseline total cholesterol levels and myocardial infarctions among middle-aged men in the United States shows that the risk of MI in men with cholesterol levels ≥ 240 mg/dL is approximately 4 times that of men with cholesterol levels < 200 mg/dL [Stamler et al. *JAMA* 2000]. The mean cholesterol level of subjects in this study was 189.9 mg/dL. Thus, you can assume the odds ratio for an MI in men with cholesterol levels ≥ 240 mg/dL compared to the average man is 4.0.

Again, Patient B's genotype at rs2383207 is "A/A" and $P(D \mid \text{"A/A"}) = 0.175$, and the average man has $P(D) = 0.212$. Integrate patient B's genetic risk with his environmental risk. Assuming each risk factor is statistically independent, what is patient B's risk of getting a heart attack, taking into account his genotype at rs2383207, and his cholesterol level of 250 mg/dL?

(a) 45.9%
(b) 52.6%
(c) 70.0%
(d) 84.8%
(e) I do not know how to calculate this

7. List at least 3 reasons why using one's genotype at rs2383207 to assess risk of a myocardial infarction can be problematic:

- rs238207 has modest odds ratio for MI
- Family history is stronger predictor than SNPs for genetic risk
- Only one SNP being used to assess risk, does not capture all of heritability
- rs2383207 is not the GWAS-identified SNP or disease-causing, and linkage disequilibrium blocks may differ from population to population
- Non-genetic risk factors play a large role in one's risk for MI

Genetics 210 Post-Course Survey (Genotype Data)

Section IV: Attitudes and reflections on personal genotyping experience

1. Which of the following reasons compelled you to undergo personal genotyping (check all that apply):

- ☐ General curiosity about my genetic makeup
- ☐ To see if a specific disease runs in my family or is in my DNA
- ☐ To learn about my genetic makeup without going through a physician
- ☐ Would use it to provide information about a family member's health risk
- ☐ To help me understand what patients learn/experience
- ☐ To help me understand principles of human genetics
- ☐ Other ________________________

Of these reasons, which was the *most* compelling (choose **one**):

- ☐ General curiosity about my genetic makeup
- ☐ To see if a specific disease runs in my family or is in my DNA
- ☐ To learn about my genetic makeup without going through a physician
- ☐ Would use it to provide information about a family member's health risk
- ☐ To help me understand what patients learn/experience
- ☐ To help me understand principles of human genetics
- ☐ Other ________________________

2. With respect to your feelings towards undergoing personal genotyping, which of the following best reflects your beliefs:

- ☐ I have significant regret that I underwent it
- ☐ I have mild regret that I underwent it
- ☐ My feelings are neutral
- ☐ I am mildly pleased that I underwent it
- ☐ I am significantly pleased that I underwent it

3. What was the greatest benefit you experienced by undergoing personal genotyping? (required comment box)

4. What was the greatest downside you experienced by undergoing personal genotyping? (required comment box)

5. I experienced anxiety when deciding whether to undergo personal genotyping

☐ Strongly disagree ☐ Disagree ☐ Neither agree nor disagree ☐ Agree ☐ Strongly agree

6. I experienced anxiety when awaiting my personal genotyping test results

☐ Strongly disagree ☐ Disagree ☐ Neither agree nor disagree ☐ Agree ☐ Strongly agree

Genetics 210 Post-Course Survey (Genotype Data)

7. I experienced anxiety after I received my personal genotyping test results

☐ Strongly disagree ☐ Disagree ☐ Neither agree nor disagree ☐ Agree ☐ Strongly agree

8. I believe the opportunity to ask a healthcare professional (e.g. genetic counselor, medical geneticist, or other physicians) for help in interpreting the results is an important component to a personal genotyping offer.

☐ Strongly disagree ☐ Disagree ☐ Neither agree nor disagree ☐ Agree ☐ Strongly agree

9. Have you or do you plan to ask a healthcare professional for help in interpreting your genotyping results?

☐ Yes, I already have ☐ Yes, I plan to ☐ No, I do not plan to

If you answered **yes**, which type(s) of healthcare provider (check all that apply):

☐ My primary physician (internist)
☐ A subspecialty physician (e.g. cardiologist, neurologist)
☐ A geneticist
☐ A genetic counselor

And what type of information have you or do you plan to share?

☐ All of my results
☐ Only results of specific high-risk alleles
☐ Only results of specific low-risk alleles
☐ Only pharmacogenetic testing results
☐ Only results related to condition(s) known in my family history

10. Please check any of the following actions that you have taken *specifically* as a result of receiving your genotyping results:

☐ Set up or attended an appointment with my primary care doctor
☐ Cancelled an appointment with my primary care doctor because of my decreased risks
☐ Set up or attended an appointment with a specialist doctor to discuss a condition I am at high risk to develop
☐ Set up an appointment for or underwent a screening test that I would not otherwise have had
☐ Cancelled an appointment for a screening test because of my decreased risks
☐ Discussed a medication change with my physician
☐ Enacted a medication change after talking with my physician
☐ Quit smoking
☐ Changed my diet in a positive manner due to elevated risks
☐ Changed my diet in a negative manner due to decreased risks
☐ Increased my exercise habits

☐ Begun taking vitamins and/or homeopathic remedies
☐ Contemplated diet, exercise or smoking habits, but have not yet made changes
☐ Performed internet research on the condition for which I am at risk
☐ Talked with family members about my genotyping results
☐ Talked with family members to learn more about my family history
☐ I have not undertaken any of these actions

11. For the actions you selected in the previous question, which of the following best describes your thoughts about it prior to genotyping:

☐ The action(s) were things that I was already planning to do, or was actively doing
☐ The action(s) were things that I had previously attempted and the genotyping results moved me to try again
☐ The action(s) were things that I had previously contemplated but not attempted, and the genotyping results moved me to attempt them
☐ The action(s) were not things that I had previously contemplated and the genotyping results moved me to contemplate them
☐ I am in the process of collecting more information to try to decide what actions, if any, are appropriate
☐ I have not taken any actions specifically as a result of my genotyping results

12. On the basis of my own experience, I would recommend personal genotyping to others

☐ Strongly disagree ☐ Disagree ☐ Neither agree nor disagree ☐ Agree ☐ Strongly agree

13. I would get my genome sequenced once it becomes affordable to me.

☐ Strongly disagree ☐ Disagree ☐ Neither agree nor disagree ☐ Agree ☐ Strongly agree

14. I believe I have a better understanding of principles of human genetics on the basis of having undergone personal genotyping

☐ Strongly disagree ☐ Disagree ☐ Neither agree nor disagree ☐ Agree ☐ Strongly agree

15. Undergoing personal genotyping was an important part of my learning in Genetics 210

☐ Strongly disagree ☐ Disagree ☐ Neither agree nor disagree ☐ Agree ☐ Strongly agree

16. I would have learned just as much from Genetics 210 had I *not* undergone personal genotyping and used only publicly available genotype data.

☐ Strongly disagree ☐ Disagree ☐ Neither agree nor disagree ☐ Agree ☐ Strongly agree

17. This course helped me understand what a patient's experience might be like if they chose to undergo personal genotyping.

Genetics 210 Post-Course Survey (Genotype Data)

☐ Strongly disagree ☐ Disagree ☐ Neither agree nor disagree ☐ Agree ☐ Strongly agree

18. I was able to easily go back and forth between my personal genotype dataset and the provided publicly available dataset as I worked on data analysis exercises.

☐ Strongly disagree ☐ Disagree ☐ Neither agree nor disagree ☐ Agree ☐ Strongly agree

19. I felt that the professors knew whether I had undergone personal genotyping.

☐ Strongly disagree ☐ Disagree ☐ Neither agree nor disagree ☐ Agree ☐ Strongly agree

20. I felt that I was at a disadvantage in the class because the professors knew whether I had undergone personal genotyping.

☐ Strongly disagree ☐ Disagree ☐ Neither agree nor disagree ☐ Agree ☐ Strongly agree

21. I felt required to divulge my genotype information in order to ask questions of the professors in Genetics 210.

☐ Strongly disagree ☐ Disagree ☐ Neither agree nor disagree ☐ Agree ☐ Strongly agree

22. I felt comfortable divulging my genotype information in order to ask questions of the professors in Genetics 210.

☐ Strongly disagree ☐ Disagree ☐ Neither agree nor disagree ☐ Agree ☐ Strongly agree

23. With regards to the offer of personal genotyping as part of a curriculum, which of the following best reflects your beliefs:

☐ Personal genotyping should be required for all medical students and graduate students in the core curriculum (GENE 202/203)
☐ Personal genotyping should be made available on an optional basis for all medical students and graduate students in the core curriculum (GENE 202/203)
☐ Personal genotyping should be made available on an optional basis for medical students and graduate students in elective classes such as GENE 210
☐ Personal genotyping should not be made available as part of a course, but could be offered to students in an optional manner through other venues
☐ Personal genotyping should not be made available to students in any formal manner.
